# Preparation of Nano-TiO_2_-Coated SiO_2_ Microsphere Composite Material and Evaluation of Its Self-Cleaning Property

**DOI:** 10.3390/nano7110367

**Published:** 2017-11-03

**Authors:** Sijia Sun, Tongrong Deng, Hao Ding, Ying Chen, Wanting Chen

**Affiliations:** Beijing Key Laboratory of Materials Utilization of Nonmetallic Minerals and Solid Wastes, National Laboratory of Mineral Materials, School of Materials Science and Technology, China University of Geosciences, Xueyuan Road, Haidian District, Beijing 100083, China; ssjcugb@163.com (S.S.); 18010157480@163.com (T.D.); chenying@cugb.edu.cn (Y.C.); wantingchen123@163.com (W.C.)

**Keywords:** SiO_2_, nano-TiO_2_, self-cleaning, hydrophilicity

## Abstract

In order to improve the dispersion of nano-TiO_2_ particles and enhance its self-cleaning properties, including photocatalytic degradation of pollutants and surface hydrophilicity, we prepared nano-TiO_2_-coated SiO_2_ microsphere composite self-cleaning materials (SiO_2_–TiO_2_) by co-grinding SiO_2_ microspheres and TiO_2_ soliquid and calcining the ground product. The structure, morphology, and self-cleaning properties of the SiO_2_–TiO_2_ were characterized. The characterization results showed that the degradation efficiency of methyl orange by SiO_2_–TiO_2_ was 97%, which was significantly higher than that obtained by pure nano-TiO_2_. The minimum water contact angle of SiO_2_–TiO_2_ was 8°, indicating strong hydrophilicity and the good self-cleaning effect. The as-prepared SiO_2_–TiO_2_ was characterized by the nano-TiO_2_ particles uniformly coated on the SiO_2_ microspheres and distributed in the gap among the microspheres. The nano-TiO_2_ particles were in an anatase phase with the particle size of 15–20 nm. The nano-TiO_2_ particles were combined with SiO_2_ microspheres via the dehydroxylation of hydroxyl groups on their surfaces.

## 1. Introduction

Nano-titanium dioxide (TiO_2_) is a typical semiconductor material with excellent properties. Moreover, it is stable, cheap, and non-toxic [1,2]. Therefore, it has been widely applied in the environmental protection [3], energy [4], and other fields [5,6]. In addition to the photocatalytic activity of TiO_2_ under ultraviolet (UV) irradiation, the self-cleaning effect due to photoinduced hydrophilic properties of TiO_2_ has always been one of the hotspots [7,8]. Its self-cleaning mechanism is generally ascribed to two effects [9,10]. Firstly, under the irradiation of ultraviolet light or ultraviolet in sunlight, the active components induced by the photocatalytic action of TiO_2_ on the TiO_2_ self-cleaning film can react with the pollutants adhering to the surface, thus achieving the decomposition of pollutants. Secondly, due to the super-hydrophilicity of the self-cleaning film, the decomposed products can be washed away by rain, so as to maintain the clean material surface [11]. In China and other developing countries, the contents of dust and oily dirt are high in the urban atmosphere and dust and oily dirt tend to adhere to building walls and glass surface to make the surface dirty. Nano-TiO_2_ self-cleaning materials may be used to coat such surfaces [12,13].

However, some factors restrict the application scope of nano-TiO_2_ self-cleaning materials. For example, the agglomeration phenomenon and poor dispersivity of TiO_2_ particles in the application system significantly reduces its self-cleaning effect [14,15]. Coating TiO_2_ particles on the matrix surface can significantly improve the dispersibility of TiO_2_ particles and enhance the photocatalytic efficiency and self-cleaning performance under the synergistic effect of the matrix [16,17]. In this way, the aforementioned problems may be solved. Many silicon materials are used as substrates to prepare nano-TiO_2_ coated composite catalysts, such as quartz tube [18], glass fibers [19], and nano-silica [20]. These catalysts all exhibit the good photocatalytic activity with different functional characteristics. Meanwhile, the micro-nano-morphology of the carrier-based nanoparticles, which are constructed from the surface of the composite self-cleaning material, can also increase the roughness of the self-cleaning film and further improve the super-hydrophilicor super-hydrophobic properties [21,22] Prabhu [23] prepared the reduced graphene oxide (rGO)/TiO_2_ composite self-cleaning material according to the solvothermal method and improved the visible light absorption efficiency of the composite self-cleaning materials, which exhibited the good photocatalytic efficiency and super-hydrophilic performance under light irradiation. Zhou [24] added the prepared SiO_2_–TiO_2_ composite colloidal particles into the fluorocarbon coating and realized more stable self-cleaning performance than that of adding single nano-TiO_2_ particles under ultraviolet light irradiation, thus suggesting its possible industrial application in outdoor environments. Zhang [25] and Ciprian [26] prepared SiO_2_–TiO_2_ composite films by the sol-gel impregnation and freeze-drying deposition method and realized the excellent self-cleaning performance and high transmittance to visible light. In general, the abovementioned preparation methods of nano-TiO_2_ composite self-cleaning material have some problems, such as the high cost, the complicated process and the difficulty in large-scale production and application. Therefore, it is necessary to select cheap matrix materials and simple composite process. Surolia [27] prepared the TiO_2_-coated fly ash photocatalyst via the sol-gel method with the cheap fly ash as substrate, exhibiting well photocatalysis degradation performance. Therefore, it is an effective way to improve the efficiency of resource utilization by using natural mineral or industrial by-product as substrate to prepare nano-TiO_2_-coated photocatalytic material. 

In this study, with SiO_2_ microspheres as the matrix, nano-TiO_2_-coated SiO_2_ microsphere composite self-cleaning materials (SiO_2_–TiO_2_) were prepared by the wet grinding of SiO_2_ microspheres and nano-TiO_2_ soliquid and the subsequent calcination of the ground product. Then, we determined the photocatalytic activity and photoinduced hydrophilicity of SiO_2_–TiO_2_, analyzed the structure and morphology, and discussed the mechanism of the interaction between TiO_2_ and SiO_2_ particles. The SiO_2_ microspheres used in this study were recovered from the by-product, silica fume, which was produced during the industrial production of fused zirconia. The SiO_2_ microspheres mainly exist in the amorphous phase and have regular morphology, high surface activity, and low cost [28,29]. However, during the past years, silica fume was usually applied in cement, concrete and refractory products as an additive and its use efficiency was low [30,31]. To the best of our knowledge, the preparation of functional materials including composite photocatalytic materials with SiO_2_ microsphere as a matrix was seldom reported. In the study, the spherical shape of the SiO_2_ microspheres can increase the fluidity of SiO_2_–TiO_2_ and promote the film formation process and the micrometer size of the SiO_2_ can improve the recyclability of nano-TiO_2_. It is expected that the SiO_2_ microspheres can exert a synergistic effect on the performance of SiO_2_–TiO_2_ and reduce the cost of composite self-cleaning materials [25]. Meanwhile, the mechanical-chemical grinding method used in this study is a simple and non-pollution particle compound method. We prepared the SiO_2_–TiO_2_ composite materials with the good photocatalysis activity and self-cleaning effect via a simple composite process with cheap matrix materials. The preparation process exhibits significant economic and environmental values. 

## 2. Methods

### 2.1. Raw Materials and Reagents

The SiO_2_ microspheres used in this study were recovered from the by-product, silica fume, which was produced during the industrial production of fused zirconia and was provided by a zirconia production enterprise in Jiaozuo (Jiaozuo, China). The main chemical constituents (mass fraction, %) of SiO_2_ microspheres were 93.78% SiO_2_ and 4.96% ZrO_2_. SiO_2_ is mainly composed of amorphous phase, exhibiting the microsphere morphology with the particle size of 1–3 μm. The SiO_2_ particles are aggregated to form the aggregates with the larger particle size. After depolymerizing the aggregates, the SiO_2_ microspheres exist in a dispersed state. 

Tetrabutyl titanate (C_16_H_36_O_4_Ti) from Beijing Chemical Industry Group Co., Ltd. (Beijing, China) was used as the titanium source. Acetylacetone (C_5_H_8_O_2_) supplied by Xi Long Chemical Co., Ltd. (Guangzhou, China) was used as a hydrolysis control agent. Methyl orange (C_14_H_14_N_3_SO_3_Na) from Beijing Chemical Industry Group Co., Ltd. (Beijing, China) was used as a target pollution for photocatalytic degradation. Ethanol and deionized water are also used as solvents throughout the preparation process.

### 2.2. Preparation Method

#### 2.2.1. Depolymerization of SiO_2_ Microspheres

Considering the agglomeration effect of particles in the raw SiO_2_ microspheres, SiO_2_ microspheres need to be depolymerized and dispersed before compositing with nano-TiO_2_. The depolymerization method was described as follows: The SiO_2_ microsphere materials were added into the ethanol solution to form a suspension. After adding ceramic grinding balls (the ratio of ball to material, 3:1), the suspension was then ground in the mixing mill (CSDM-S3, Beijing Paleozoic Powder Technology Co., Ltd., Beijing, China) for 60 min. Finally, the dispersed SiO_2_ microspheres were obtained after ball-material separation, filtration, and desiccation.

#### 2.2.2. Preparation of Nano-TiO_2_ Soliquid

Firstly, 8.5 mL of tetrabutyl titanate was dissolved into 10 mL of ethanol solution. The mixed solution was stirred evenly and marked as Solution A. Then, 1.3 mL of acetylacetone was dissolved into 10 mL of ethanol solution, and the obtained solution was marked as Solution B. Then, Solution B was slowly added into Solution A and 19.35 mL of the mixture of ethanol and water (water 0.85 mL) was also added into Solution A. Afterwards, the mixture was stirred vigorously at room temperature for 12 h and the stirred mixture was aged for 48 h to obtain the nano-TiO_2_ soliquid. The viscosity of the nano-TiO_2_ soliquid obtained after 48-h aging was measured to be 2 × 10^−3^ Pa·s by a digital display viscometer (NDJ-8S, Shanghai Precision Instrument and Meter Co., Ltd., Shanghai, China). For comparison, partial nano-TiO_2_ soliquid was dried and calcined to prepare TiO_2_ nanoparticles. According to the X-ray diffraction (XRD) data and the Scherrer Equation, the grain size of nano-TiO_2_ was calculated to be 15–20 nm.

#### 2.2.3. Preparation of SiO_2_–TiO_2_

Firstly, the dispersed SiO_2_ microspheres were added into the ethanol solution, which was stirred to form a suspension. Secondly, the suspension was added into the aged nano-TiO_2_ soliquid to form the SiO_2_/TiO_2_ mixture. Thirdly, the SiO_2_/TiO_2_ mixture were stirred by a CSDM-S3 mixing mill (Beijing Gosdel Powder&Technology Co., Ltd., Beijing, China) for 90 min after the addition of a certain amount of grinding balls to obtain the SiO_2_/TiO_2_ soliquid composites. Then, the SiO_2_/TiO_2_ soliquid composites were put in a SRJX-5-13 chamber electric furnace (Tianjin Taisite Instrument Co., LTD, Tianjin, China) and calcined at 500 °C for 2 h. Finally, the SiO_2_–TiO_2_ was prepared.

### 2.3. Characterization

#### 2.3.1. Evaluation of Self-Cleaning Performance

##### Photocatalytic Activity

The photocatalytic degradation performance of SiO_2_–TiO_2_ was tested with the methyl orange as the target degradation pollutant. The system was irradiated by a mercury lamp (100 W, the main wavelength of 254 nm). Then, 40 mg of SiO_2_–TiO_2_ was added to 50 mL of prepared methyl orange dilution (concentration 10 mg/L). In order to reduce the measurement error caused by sample adsorption, the dark reaction was carried out for 0.5 h and then the concentration of methyl orange (*C*_0_) in the solution was measured. After turning on the light source, the concentration of methyl orange (*C*) in solution was measured every 20 min. The photocatalytic degradation performance of the samples was characterized and evaluated based on the change of *C*/*C*_0_.

The concentration of methyl orange was measured according to the following procedure. Firstly, the solution was centrifuged and the absorbance of the supernatant was measured with a Cary 5000 UV–VIS spectrophotometer (USA Varian, Palo Alto, CA, USA). The concentration of methyl orange in the solution was calculated according to the relationship between absorbance and concentration.

##### Hydrophilicity

The hydrophilicity of the SiO_2_–TiO_2_ particles was characterized based on the wetting degree of water on its surface. The wetting degree was reflected by the measured water contact angle on its surface. The SiO_2_–TiO_2_ composite powder was pressed into a sheet-like sample by a tableting machine and then the water contact angle was measured by a contact angle meter (JC2000D, Shanghai Zhongchen Digital Technic Apparatus Co. Ltd., Shanghai, China) three times. The measurement results were averaged.

#### 2.3.2. Characterization of Structure and Morphology

We observed the morphology of SiO_2_–TiO_2_ by scanning electron microscope (SEM) (S-3500N, Hitachi, Ltd., Tokyo, Japan) and transmission electron microscope (TEM) (FEI Tecnai G2 F20, Portland, OR, USA). The surface functional groups were examined by an infrared spectroscope (Spectrum 100, PerkinElmer Instruments (Shanghai) Co., Ltd., Shanghai, China) with KBr as the medium, and the weights of each sample and KBr were, respectively, 1 and 200 mg. The phase analysis was carried out with an X-ray diffractometer (D/MAX2000, Rigaku Corporation, Tokyo, Japan).The specific surface areas of SiO_2_ and SiO_2_–TiO_2_ were tested by the QuadraSorb SI specific surface area analyzer (Quantachrome Instrument Company, Boynton Beach, FL, USA). In addition, the surface roughness of SiO_2_ microspheres and SiO_2_–TiO_2_ were evaluated using a Mutimode VIII atomic force microscope (Bruke, Fremont, CA, USA).

## 3. Results and Discussion

### 3.1. Properties of SiO_2_–TiO_2_

#### 3.1.1. Photocatalytic Properties of SiO_2_–TiO_2_

**Figure 1:**
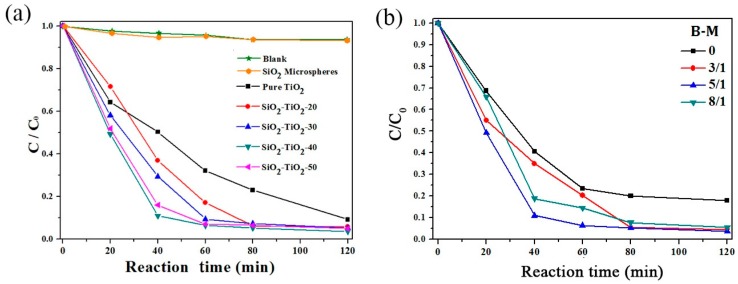
Influences of (**a**) TiO_2_ ratio and (**b**) B-M ratio on the photocatalytic performance of SiO_2_–TiO_2_. (**a**) SiO_2_–TiO_2_-20, 30, 40, 50 represent the mass ratio of TiO_2_ to SiO_2_–TiO_2_ is 20%, 30%, 40% and 50%; and (**b**) B-M represents the mass ratio of grinding balls to the materials.

Figure 1a represents the degradation behaviors of methyl orange dye during irradiation as a function of time (min) in the presence of SiO_2_–TiO_2_ with different TiO_2_ ratios (the mass ratio of TiO_2_ to SiO_2_–TiO_2_). As shown in Figure 1a, the SiO_2_ microspheres exhibit no degradation effect on methyl orange, whereas pure TiO_2_ has a certain degradation effect on methyl orange. All of the prepared SiO_2_–TiO_2_ materials exhibit the significantly higher photocatalytic degradation efficiency on methyl orange dye than that of pure nano-TiO_2_. Among these SiO_2_–TiO_2_ samples, with SiO_2_–TiO_2_-40 (TiO_2_ ratio is 40%) as the photocatalyst, after the solution was irradiated for 40 min, the *C*/*Co* was reduced to about 0.1 and the degradation efficiency reached 90%. After the 120 min irradiation, the degradation efficiency reached 97%. With the pure nano-TiO_2_ as the photocatalyst, the degradation efficiencies after 40 and 120 min respectively reached 50% and 90%. The abovementioned results indicated that the photocatalytic activity of nano-TiO_2_ had been greatly improved when TiO_2_ coated the surface of SiO_2_ microspheres. In addition, the TiO_2_ ratio had a significant effect on the degradation efficiency of SiO_2_–TiO_2_. With the increase in the TiO_2_ ratio from 20% to 40%, the photocatalytic degradation efficiency gradually increased and finally reached its maximum value. When the mass ratio of TiO_2_ increased to 50%, the degradation efficiency decreased. However, the degradation efficiency of SiO_2_–TiO_2_ with different TiO_2_ ratios was always higher than that of pure nano-TiO_2_. The phenomenon might be interpreted in two aspects: Firstly, the coating of nano-TiO_2_ on SiO_2_ microsphere surface could improve the dispersibility of nano-TiO_2_, thus resulting in an increase in the number of reactive groups under irradiation and increasing the quantum efficiency. Secondly, SiO_2_ had a high reflection efficiency on ultraviolet radiation, and the light reflected by SiO_2_ could be absorbed by TiO_2_, thus improving the absorption of ultraviolet light by SiO_2_–TiO_2_. The specific surface area analysis results showed that the surface area of SiO_2_ had been significantly incresed from its original value of 5.698 to 44.410 m^2^/g after TiO_2_ coating. This result also comfirmed that the SiO_2_ microspheres had been coated by nano TiO_2_ effectively.Figure 1b shows the influence of the ratio of grinding ball to materials (B-M) in the grinding process on the photocatalytic activity of SiO_2_–TiO_2_. The degradation efficiency of SiO_2_–TiO_2_ samples prepared with grinding balls was significantly higher than that of the SiO_2_–TiO_2_ prepared without grinding balls (B-M is 0). The degradation effect was the best when the B-M ratio was 5. After 120 min irradiation, the highest degradation efficiency was 95% (*C*/*C*_0_ = 0.05) at the B-M ratio of 5% and 65% at the B-M of 0. The above results showed that the grinding process had an important effect on the performance of SiO_2_–TiO_2_. Therefore, the proper B-M ratio should be selected. As shown in Figure 1b, the degradation effect of SiO_2_–TiO_2_ is stronger than that of pure nano-TiO_2_. The result is consistent with the results shown in Figure 1a.

**Figure 2:**
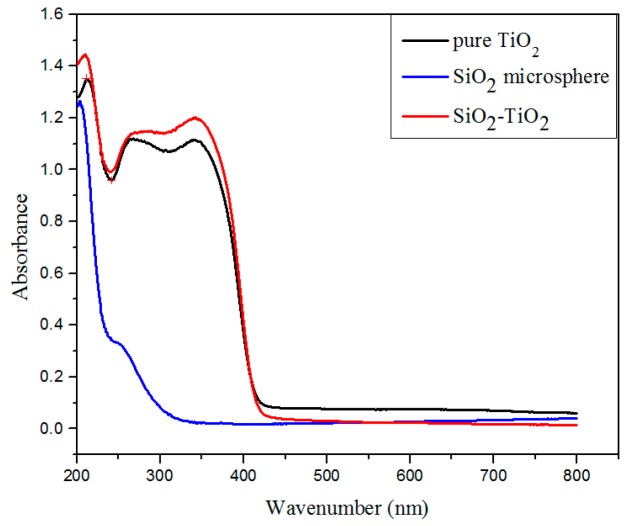
UV–VIS absorption spectra of pure TiO_2_, SiO_2_ microsphere and SiO_2_–TiO_2_.

The UV–VIS absorption spectra of bare SiO_2_ microspheres, nano-TiO_2_, and SiO_2_–TiO_2_-50 were obtained for comparison (Figure 2). The light absorption of SiO_2_ in a wavelength range between 300 and 400 nm was insignificant, whereas TiO_2_ absorbed light with the wavelength below 400 nm. The SiO_2_–TiO_2_ exhibited the higher light absorption in a wavelength range from 200 to 400 nm than that of pure nano-TiO_2_, which was completely different from bare SiO_2_ microspheres. The results indicated that the SiO_2_–TiO_2_ had the higher UV absorption due to the high reflection efficiency on ultraviolet radiation by SiO_2_ microspheres, confirming that SiO_2_ microspheres were coated by nano-TiO_2_ particles with similar light absorption properties to TiO_2_. Meanwhile, this results contribute to the good photocatalytic activity of SiO_2_–TiO_2_.

#### 3.1.2. Hydrophilic Properties of SiO_2_–TiO_2_

**Figure 3:**
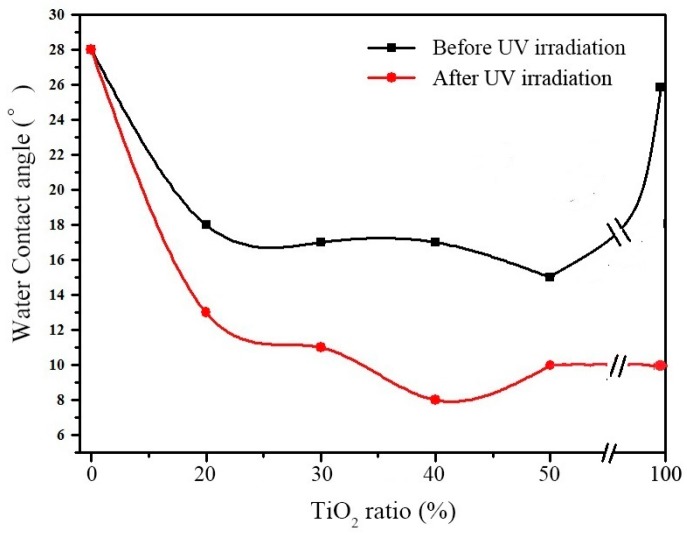
Relationship between the water contact angle and the content of TiO_2_.

Figure 3 shows the change of water contact angle of SiO_2_–TiO_2_ particles with different TiO_2_ ratios after irradiation by ultraviolet light for 2 h. For the SiO_2_ microsphere materials, the contact angle was maintained to be 28° after UV irradiation, indicating that the UV light had no effect on its hydrophilicity. The water contact angle of pure TiO_2_ is 26° before UV irradiation, which is higher than that of SiO_2_–TiO_2_, indicating that the coating of TiO_2_ on SiO_2_ surface can improve the hydrophilicity of TiO_2_. The improvement effect may be interpreted as follows. The dispersion of nano-TiO_2_ was improved and then more active hydroxyl groups on TiO_2_ surface were exposed. Meanwhile, the water contact angle of pure TiO_2_ decreased from 26° to 10° after UV irradiation, indicating the photoinduced hydrophilicity of TiO_2_. The water contact angle of SiO_2_–TiO_2_ was 15–18° and decreased to 8–13° after UV irradiation, showing the strong hydrophilicity. The SiO_2_–TiO_2_-40 (TiO_2_ ratio is 40) showed the strongest hydrophilicity and its water contact angles were 17° and 8° before and after UV irradiation respectively. The strong photo-induced hydrophilicity and photocatalytic activity of SiO_2_–TiO_2_ indicate its good self-cleaning performance.

**Figure 4:**
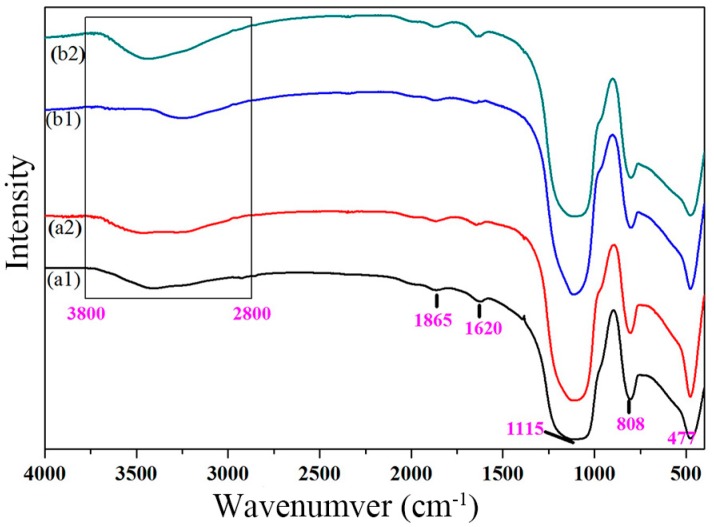
Fourier transform infrared spectroscopy (FT-IR) spectrum of the SiO_2_–TiO_2_ with different TiO_2_ ratios. (**a1**) SiO_2_–TiO_2_-20, before UV irradiation; (**a2**) SiO_2_–TiO_2_-20, after UV irradiation; (**b1**) SiO_2_–TiO_2_-30, before UV irradiation; and (**b2**) SiO_2_–TiO_2_-30, after UV irradiation; The black rectangle region represents the absorption bands caused by the vibration of the hydroxyl radical

To investigate the mechanism of the photoinduced hydrophilicity of SiO_2_–TiO_2_, the infrared spectral analysis was carried out. Figure 4 shows the Fourier transform infrared spectroscopy (FT-IR) spectra of SiO_2_–TiO_2_-20 and SiO_2_–TiO_2_-30 before and after UV irradiation. The characteristic absorption peaks in the range of 2800–3800 cm^−1^ and 1620 cm^−1^ in all the samples were ascribed to the vibration of the hydroxyl groups on the SiO_2_–TiO_2_ surface. When the TiO_2_ ratio was 30%, after the UV irradiation (b2 in Figure 4), the intensity of the absorption peak in the range of 2800–3800 cm^−1^ in the FTIR spectrum of SiO_2_–TiO_2_ was higher than that in the spectrum b1 (before the UV irradiation) and the peak was shifted to the higher wavenumber. Meanwhile, the absorption peak at 1620 cm^−1^ in b2 was sharper than that in b1. The abovementioned results indicated that the number of hydroxyl groups on the surface of SiO_2_–TiO_2_ increased after UV irradiation and that the SiO_2_–TiO_2_ exhibited the reaction activity with water. We believed that the production of hydroxyl groups was induced by the photoinduced action of TiO_2_. The change was consistent with the remarkable enhancement of the surface hydrophilicity of SiO_2_–TiO_2_ after UV irradiation in Figure 3.

### 3.2. Structure and Morphology of SiO_2_–TiO_2_

#### 3.2.1. XRD Analysis

**Figure 5:**
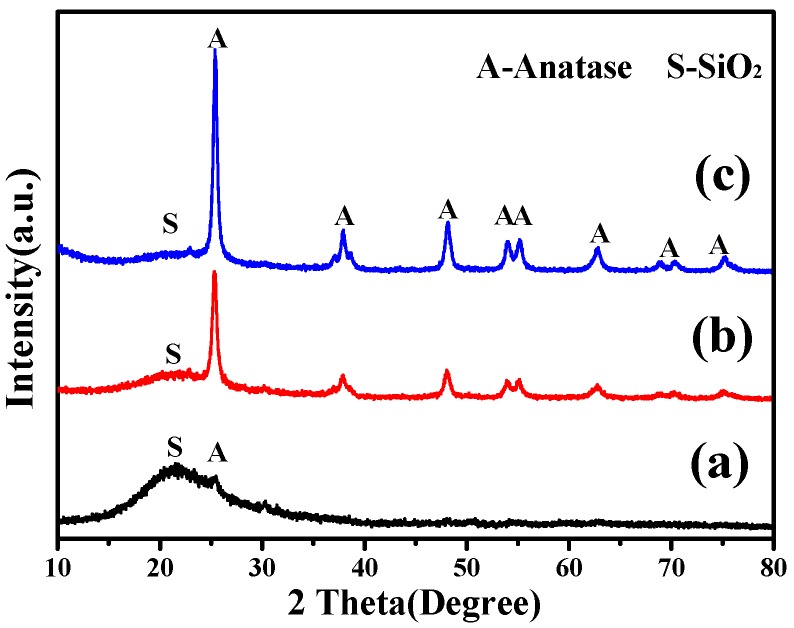
XRD patterns of SiO_2_–TiO_2_ with different TiO_2_ ratios. (**a**) 30% TiO_2_; (**b**) 40% TiO_2_; and (**c**) 50% TiO_2_.

Figure 5 shows the XRD patterns of SiO_2_–TiO_2_ with different TiO_2_ ratios. In addition to the diffraction peak of amorphous SiO_2_ microspheres, the diffraction peaks of the anatase phase also appeared in the XRD patterns of all SiO_2_–TiO_2_ samples, and the intensity of diffraction peaks of the anatase phase increased with the increase in the TiO_2_ ratio. Especially, when the TiO_2_ ratio was 50%, the complete anatase diffraction peak (JCPDS 21-1272) appeared in the XRD pattern of SiO_2_–TiO_2_-50 (Figure 5c) [32]. The abovementioned results indicated that nano-TiO_2_ existed as an anatase phase. Among all the TiO_2_ crystal phases, the anatase exhibited the highest photocatalytic activity, which was consistent with the results of photocatalytic activity and photoinduced hydrophilicity of SiO_2_–TiO_2_.

#### 3.2.2. Morphology and Element Analysis

**Figure 6:**
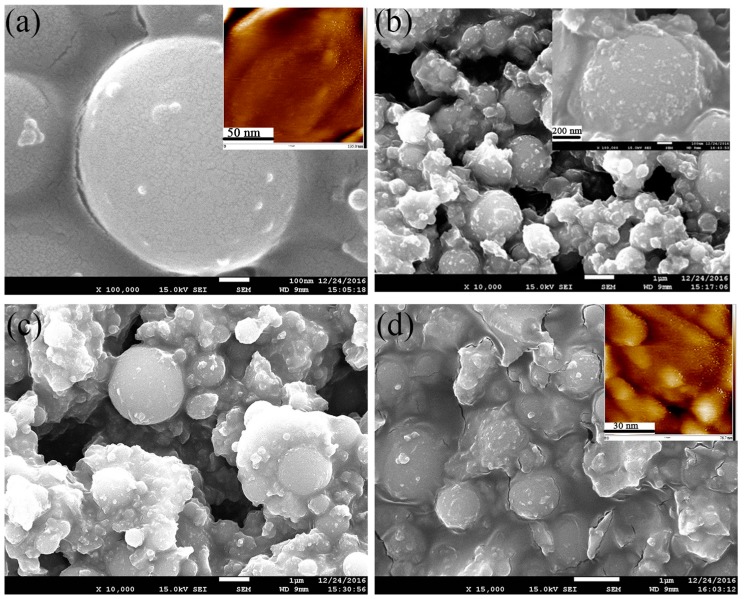
Scanning electron microscope (SEM) and atomic force microscope (AFM) images of (**a**) SiO_2_ microsphere and (**b**–**d**) SiO_2_–TiO_2_ with different ratios. (**b**) 30% TiO_2_, and the inset image is a high magnification image; (**c**) 40% TiO_2_; and (**d**) 50% TiO_2_; the inset images in (**a**,**d**) are AFM images.

Figure 6 shows the SEM images of SiO_2_–TiO_2_ with different TiO_2_ ratios. In Figure 6a, the exposed surfaces of SiO_2_ microspheres are smooth without covering. However, the micron-submicron hierarchical structure morphology can be observed in Figure 6b–d. The surface of the SiO_2_ microspheres became rough and was covered with a certain amount of irregular particles. Meanwhile, with the increase in the TiO_2_ ratio, the roughness and coverage area of the SiO_2_ microsphere surface increased accordingly. According to the preparation process, it was presumed that the coating on the surface of the microspheres should be nano-TiO_2_ particles. The surface roughness of SiO_2_ microspheres and SiO_2_–TiO_2_-50 were evaluated using an atomic force microscope, and the corresponding atomic force microscope (AFM) images were shown in Figure 6a,d (see the built-in images). The tested surface roughness of SiO_2_ microspheres and SiO_2_–TiO_2_ were 1.63 and 18.4 nm, respectively. These results show that the surface roughness of SiO_2_ increased significantly after it was coated by nano-TiO_2_, indicating that the surface structure of SiO_2_ has changed. Additionally, in the magnification image of SiO_2_–TiO_2_ shown in Figure 6b, the nano-TiO_2_ particles not only uniformly coated the surface of the SiO_2_ microspheres, but also exist in the gap among SiO_2_ microspheres. In this way, several microspheres were connected together as a whole.

**Figure 7:**
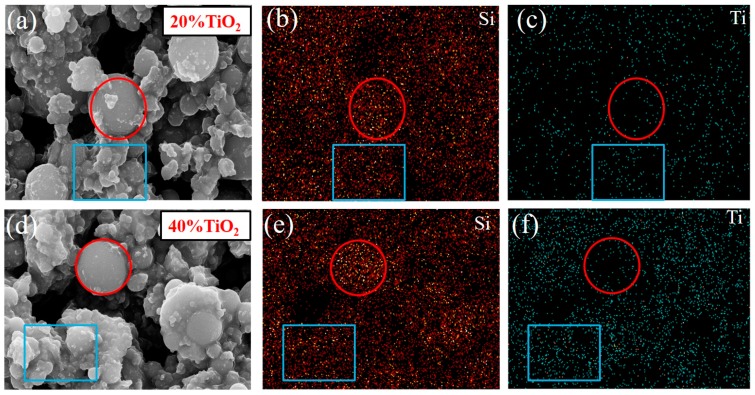
Scanning results of surface elements of SiO_2_–TiO_2_ with (**a**–**c**) 20% TiO_2_ and (**d**–**f**) 40% TiO_2_.

To confirm the composition of the coating on the surface of SiO_2_ microsphere, a surface scanning analysis of the main elements in the selected part of the SiO_2_–TiO_2_ SEM was carried out (Figure 7). The Ti element was almost distributed throughout the scan area, like the distribution of Si element. The distribution density of Ti element is proportional to the TiO_2_ ratio. This confirmed that the nano-TiO_2_ particles had coated the surface and were distributed in the gap among SiO_2_ microsphere. The results were consistent with SEM results (Figure 6).

**Figure 8:**
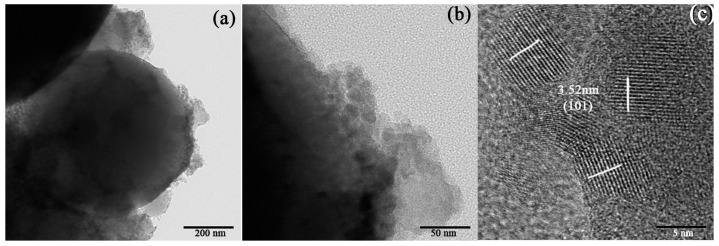
(**a**,**b**) Transmission electron microscope (TEM) and (**c**) high resolution transmission electron microscopy (HRTEM) images of SiO_2_–TiO_2_ at different scales.

Figure 8 shows the TEM and high resolution transmission electron microscopy (HRTEM) images of the SiO_2_–TiO_2_ samples (TiO_2_ ratio is 40%). Circular SiO_2_ microspheres and irregular nano-TiO_2_ particles surrounding the SiO_2_ microspheres are observed in Figure 8a, confirming that the nano-TiO_2_ particles has coated the surface of SiO_2_ microspheres. In the HRTEM (Figure 8c), the interplanar spacing of the three major facets were measured to be *d* = 0.352 nm [33], which was consistent with the (101) crystal face of anatase (JCPDS 21-1272). The above results indicated that the nano-TiO_2_ coating on the surface of SiO_2_ microspheres was anatase and that the mainly exposed crystal face was (101).

### 3.3. Mechanism of the Interaction between SiO_2_ and TiO_2_ Particles

**Figure 9:**
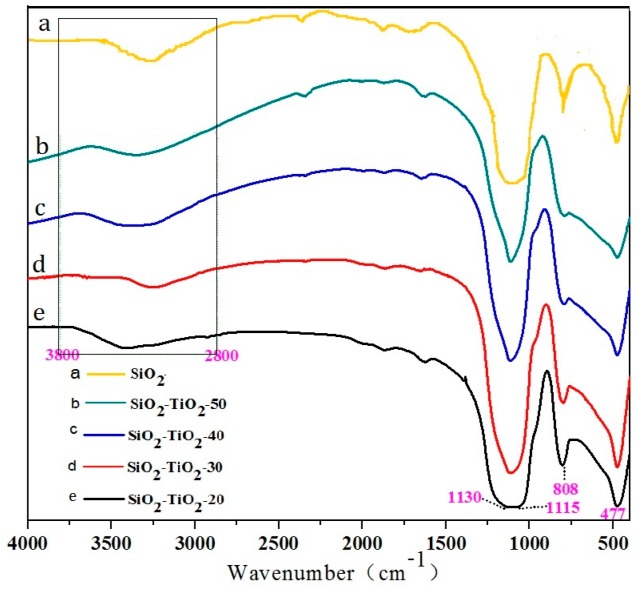
FT-IR of SiO_2_–TiO_2_ with different TiO_2_ ratios. SiO_2_–TiO_2_-20, 30, 40, 50 represent the mass ratio of TiO_2_ to SiO_2_–TiO_2_ is 20%, 30%, 40% and 50%; The black rectangle region represents the absorption peak caused by the vibration of the hydroxyl radical.

Figure 9 shows the FT-IR spectra of SiO_2_ and SiO_2_–TiO_2_ with different TiO_2_ ratios. The absorption bands at 1115, 808, and 477 cm^−1^ are typical absorption bands of Si–O bonds, indicating that the main component of the composite is SiO_2_ [34].With the increase in the TiO_2_ ratio, the intensity of absorption bands corresponding to SiO_2_ decreased, indicating that the nano-TiO_2_ coated the SiO_2_ surface. In addition, the absorption bands (3200–3550 cm^−1^) derived from Si–OH and Ti–OH showed the significant displacement and broadening phenomena when the SiO_2_ was coated by the nano-TiO_2_, indicating that the chemical environment had been changed and the association degree of hydroxyl groups on particles surface had increased. It was obviously caused by the formation of hydrogen bonds between Si–OH and Ti–OH or the further dehydroxylation reaction. It should be inferred that the chemical combination between SiO_2_ microspheres and nano-TiO_2_ particles was formed through the interaction of hydroxyl groups on their surfaces. 

**Figure 10:**
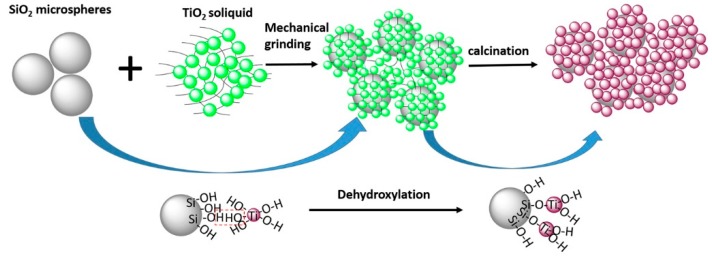
Schematic diagram of the bonding mechanism of SiO_2_–TiO_2_.

Figure 10 shows the schematic diagram of the bonding mechanism of SiO_2_–TiO_2_. Based on the above results, the bonding mechanism can be described as follows: firstly, the SiO_2_ microspheres were ground in the ethanol medium with grinding balls. The strong grinding force made SiO_2_ microspheres depolymerization and exposed more hydroxyl groups, thus displaying the higher reactivity. Secondly, the prepared nano-TiO_2_ soliquid was ground with the activated SiO_2_ violently, so that the collision probability between particles increased and lead to the contact and reactions between the hydroxyl groups on the SiO_2_ and TiO_2_ surfaces. Finally, water produced by the dehydroxylation of the particles was further removed by calcination. The SiO_2_ and TiO_2_ particles were bounded by –Si–O–Ti– bonds. The strength of the chemical bond was stronger than that of van der Waals forces and other physical forces, so the coating of nano-TiO_2_ on SiO_2_ surface was firm. 

## 4. Conclusions

In the study, with the by-product SiO_2_ microspheres produced during the industry production of fused-zirconia as the substrates, SiO_2_–TiO_2_ particles were prepared by the wet-grinding of SiO_2_ microspheres and nano-TiO_2_ and calcination of the ground product. The degradation efficiency of SiO_2_–TiO_2_ on methyl orange reached 97%, which was significantly higher than that of pure nano-TiO_2_. The water contact angle of SiO_2_–TiO_2_ was 8°, indicating the strong photoinduced hydrophilicity and the good self-cleaning effect.

The SiO_2_–TiO_2_ particles were characterized by the nano-TiO_2_ uniformly coated on the SiO_2_ microspheres and distributed in the microsphere gap. The nano-TiO_2_ particles existed in an anatase phase with the particle size of 15–20 nm and are combined with SiO_2_ microspheres by the dehydration of hydroxyl groups on particle surfaces.
